# Manifestation cutanée d'une coagulopathie intravasculaire disséminée compliquant un avortement

**DOI:** 10.11604/pamj.2014.19.273.5697

**Published:** 2014-11-12

**Authors:** Nezha Oudghiri, Mouhssine Doumiri

**Affiliations:** 1 Département d'Anesthésie Réanimation Obstétricale, Hôpital Maternité Souissi, Université Mohamed V, Rabat, Maroc

**Keywords:** avortement, coagulopathie intravasculaire disséminée, sepsis, abortion, Disseminated intravascular coagulopathy, sepsis

## Image en medicine

Patiente de 35 ans, vue aux urgences obstétricales pour troubles de la conscience et fièvre apparus deus jours après un avortement provoqué. L'examen clinique a trouvé une patiente léthargique, ictérique, dyspnéique à 26 c /min, fébrile 39,5°c, tachycarde à 120 batt /min et la pression artérielle était à 90/45 mmHg avec à l'examen gynécologique une endométrite. La radiographie du poumon et les gaz de sang étaient en faveur d'un syndrome de détresse respiratoire aigu. On a initié une réanimation armée avec oxygénothérapie, remplissage modéré,effectué des prélèvements bactériologiques et on a commencé une antibiothérapieappropriée. La biologie a montré une hyperleucocytose à 26.000 GB/mm^3^, une anémie à 9,5 g /l, une thrombopénie à 28000 /mm^3^, GOT à 837 UI /L, GPT à 520 UI/L, une bilirubine totale à 18mg/dl, un TP à 22%, un fibrinogène à 0,4 g /l, des produits de dégradation de fibrine à 226ug/ml, une urée à 0,93g /l, une créatinine à 22 mg/dl et un rapport Pao2/Fio2 à 180. L’évolution a été marquée par l'apparition de placards purpuriques de coagulation intravasculaire disséminée au niveau des deux pieds et dont l’échodoppler a confirmé la présence de microthombis distaux. On a mis la patiente sous anticoagulants à base d'héparine de bas poids moléculaire à raison de 0,4 ml par jour en sous cutané. Cet avortement a été compliqué de sepsis grave avec manifestation cutanée, rénale, hématologique, hépatique, respiratoire et neurologique. La patiente a bien évolué, et est sortie autonome de la réanimation après dix jours d'hospitalisation.

**Figure 1:**
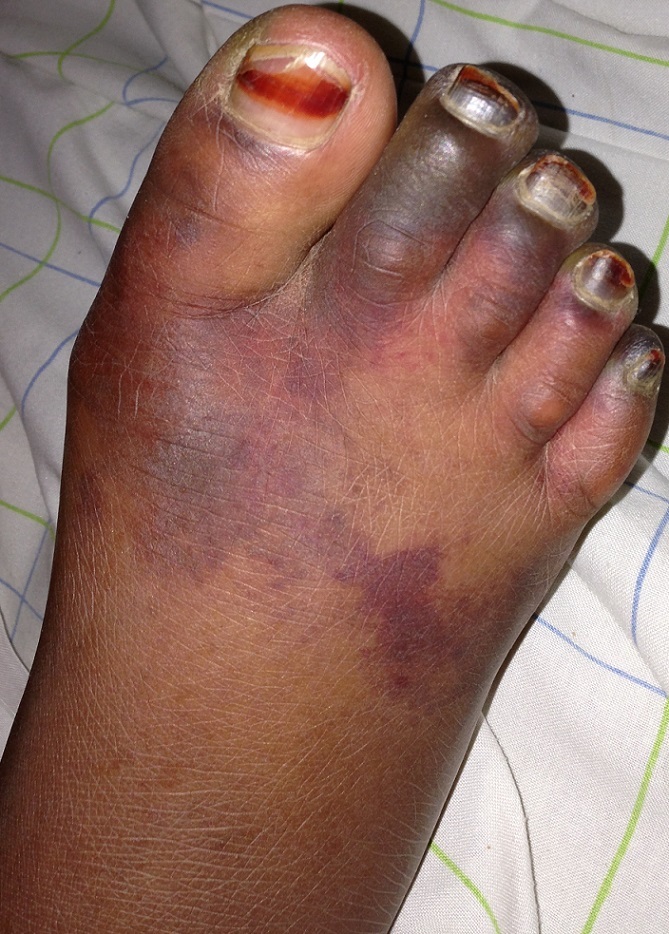
Manifestation cutanée d'une coagulopathie intravasculaire disséminée

